# Recurrent urinary tract infections and prophylactic antibiotic use in women: a cross-sectional study in primary care

**DOI:** 10.3399/BJGP.2024.0015

**Published:** 2024-08-20

**Authors:** Leigh Sanyaolu, Victoria Best, Rebecca Cannings-John, Fiona Wood, Adrian Edwards, Ashley Akbari, Gail Hayward, Haroon Ahmed

**Affiliations:** Division of Population Medicine and PRIME Centre Wales, Cardiff University, Cardiff.; Population Data Science, Swansea University, Swansea.; Centre for Trials Research, Cardiff University, Cardiff.; Division of Population Medicine and PRIME Centre Wales, Cardiff University, Cardiff.; Division of Population Medicine and PRIME Centre Wales, Cardiff University, Cardiff.; Population Data Science, Swansea University, Swansea.; Nuffield Department of Primary Care Health Sciences, University of Oxford, Oxford.; Division of Population Medicine and PRIME Centre Wales, Cardiff University, Cardiff.

**Keywords:** anti-infective agents, bacterial, drug resistance, electronic health records, primary health care, urinary, urinary tract infections

## Abstract

**Background:**

Despite the considerable morbidity caused by recurrent urinary tract infections (rUTIs), and the wider personal and public health implications from frequent antibiotic use, few studies adequately describe the prevalence and characteristics of women with rUTIs or those who use prophylactic antibiotics.

**Aim:**

To describe the prevalence, characteristics, and urine profiles of women with rUTIs with and without prophylactic antibiotic use in Welsh primary care.

**Design and setting:**

This was a retrospective cross-sectional study in Welsh general practice using the Secure Anonymised Information Linkage (SAIL) Databank.

**Method:**

The characteristics of women aged ≥18 years with rUTIs or using prophylactic antibiotics from 2010 to 2020, and associated urine culture results from 2015 to 2020, are described.

**Results:**

In total, 6.0% (*n* = 92 213/*N* = 1 547 919) had rUTIs, and 1.7% (*n* = 26 862/*N* = 1 547 919) were prescribed prophylactic antibiotics with the rates increasing after 57 years of age. Only 49.0% (*n* =13 149/*N* = 26 862) of users of prophylactic antibiotics met the definition of rUTIs before initiation. The study found that 80.8% (*n* = 44 947/*N* = 55 652) of women with rUTIs had a urine culture result in the preceding 12 months with high rates of resistance to trimethoprim and amoxicillin. Of women taking prophylactic antibiotics, 64.2% (*n* = 9926/*N* = 15 455) had a urine culture result before initiation and 18.5% (*n* = 320/*N* = 1730) of women prescribed trimethoprim had resistance to it on the antecedent sample.

**Conclusion:**

A substantial proportion of women had rUTIs or incident prophylactic antibiotic use. However, 64.2% (*n* = 9926/*N* = 15 455) of women had urine cultured before starting prophylaxis. There was a high proportion of cultured bacteria resistant to two antibiotics used for rUTI prevention and evidence of resistance to the prescribed antibiotic. More frequent urine cultures for rUTI diagnosis and before prophylactic antibiotic initiation could better inform antibiotic choices.

## Introduction

Urinary tract infections (UTIs) in women are common, and a proportion experience recurrent UTIs (rUTIs), defined as ≥2 UTIs in 6 months, or ≥3 in 12 months.1–3 rUTIs are a significant cause of morbidity and health service use.1,4,5 Estimates of the prevalence of rUTIs range from 3% to 44% of women, depending on the definition used and age or nationality studied.6–8 Women with rUTIs have frequent antibiotic exposure because of treatment for acute UTIs and potential long-term prophylaxis. Antibiotic exposure is a major driver of antimicrobial resistance (AMR), and antibiotic exposure for UTIs increases resistance within 2 months and persists for 12 months.^9^^,^^10^ Resistant UTIs have a greater impact on patients and are more costly to treat than susceptible infections.^11^^,^^12^

Despite the considerable morbidity caused by rUTIs, and the wider personal and public health implications from frequent antibiotic use, few studies adequately describe the prevalence and characteristics of women with rUTIs. Furthermore, it is unclear how well clinical practice aligns with current guidelines recommending urine culture for rUTI diagnosis.^1^ Understanding real-world practice related to diagnosing and treating rUTIs, a common condition seen and managed in primary care, is an important step towards improving patient care.

This study aimed to comprehensively describe the prevalence, characteristics, urine testing, and susceptibility profiles of women with rUTIs in Wales to understand current clinical practice and alignment with guidelines.

## Method

### Design

This was a cross-sectional study using anonymised individual-level, population-scale linked electronic health record data sources within the Secure Anonymised Information Linkage (SAIL) Databank, the ISO 27001-certified national trusted research environment (TRE) for Wales.^13^ All data sources within the SAIL Databank TRE are linkable following approvals using an anonymised linking field.^13^ The data sources used include:

- Welsh Longitudinal General Practice (WLGP) — primary care general practice data using Read codes, covering 86.1% of the Welsh population registered with 82.4% of Welsh general practices;^14^^,^^15^
- Patient Episode Database for Wales (PEDW) — secondary care hospital admission data using the International Classification of Disease version 10 (ICD-10);^16^
- Welsh Results Reporting Service (WRRS) — all-Wales urine specimen data from primary and secondary care; and^17^
- Welsh Demographic Service Dataset (WDSD) and Annual District Death Extract (ADDE) — demographic and death data.^18^^,^^19^

**Table:** How this fits in

Little is known about the prevalence, characteristics, urine testing, or the use of prophylactic antibiotics among women with recurrent urinary tract infections (rUTIs). This study found that 6.0% (*n* = 92 213/*N* = 1 547 919) of women had evidence of rUTIs in Wales from 2010 to 2020, and, from 2015–2020, 80.8% (*n* = 44 947/*N* = 55 652) had a urine culture result in the preceding 12 months. In total, 1.7% (*n* = 26 862/*N* = 1 547 919) of women used prophylactic antibiotics during the study period and, between 2015–2020, 64.2% (*n* = 9926/*N* = 15 455) had a urine culture result before starting prophylaxis, and of these 8.3% (*n* = 410/*N* = 4920) were resistant to the prescribed antibiotic. More frequent urine cultures in the workup of rUTI diagnosis and prophylactic antibiotic initiation could better inform antibiotic choice.

The Improving Prophylactic Antibiotic use for Recurrent urinary Tract infection (IMPART) patient and public team were involved in the design of this study.

### Population

Two cohorts were created. Cohort 1 included women who met the clinical definition of rUTIs (clinical cohort). Cohort 2 included women prescribed prophylactic antibiotics consistent with rUTI prevention (prophylaxis cohort; for definitions, see below). Eligibility criteria for the two cohorts are described in Box 1.

**Box 1.** Inclusion and exclusion criteria for study entry

**Inclusion criteria**	Sufficient data linkage quality (Supplementary Box S1) Sex recorded as female using the WDSD^19^ Aged ≥18 years and alive during the study period (1 January 2010 to 31 December 2020) Registered with a SAIL-providing general practice during the study period Registered for ≥12 months before cohort entry for women with rUTIs or registered for ≥18 months before cohort entry for women taking prophylactic antibiotics (to capture comorbidity and urine microbiology data) Met the definition of an rUTI or prophylactic antibiotic use
**Exclusion criteria**	Catheter use was recorded at any point before cohort entry Pregnancy was recorded in the 40 weeks before the cohort entry date to ensure women were not pregnant at cohort entry. Patients were eligible subsequently, provided they met the inclusion criteria and if pregnancy was not recorded within 40 weeks before the cohort entry date

*rUTI = recurrent urinary tract infection. SAIL = Secure Anonymised Information Linkage.*

### Case ascertainment

#### Definition of recurrent UTIs

In the current study, rUTIs were defined as ≥2 acute UTIs within 6 months, or ≥3 within 12 months.1 How acute UTIs were defined is shown in Box 2. Consultations and hospital admissions for acute UTIs were identified using Read and ICD-10 code lists (Supplementary Boxes S2–S4). More than one acute UTI within a 28-day period was considered a repeat consultation for the same episode. The date of the first consultation was recorded as the acute UTI date (Supplementary Figure S1). For hospital-diagnosed UTIs, the start date of the UTI was the first date of an episode containing a UTI ICD-10 code and the end date was its final date. The first time a woman met the definition of rUTI was used as the date of rUTI diagnosis.

**Box 2.** Definitions of acute UTIs^a^

**Clinical scenario**	**UTI-related Read code (WLGP data)**	**Antibiotic prescription^b^ (WLGP data)**	**UTI-related ICD-10 code (PEDW data)**	**Urine culture result (WRRS data)**	**Time period between codes**
1. General practice clinically diagnosed and treated UTI	• Yes	• Yes	• No	• No	• Same date
2. Hospital-diagnosed and treated UTI	• No	• No	• Yes	• No	• Not applicable
3. General practice clinically diagnosed, microbiologically confirmed, and treated UTI	• Yes	• Yes	• No	• Confirmed UTI	• Urine culture result within 7 days of general practice clinically diagnosed and treated UTI. Earliest code = date of UTI
4. Hospital-diagnosed, microbiologically confirmed, and treated UTI	• No	• No	• Yes	• ConfirmedUTI	• Within 7 days. Earliest code = date of UTI

a *‘Confirmed UTI’ is defined as: bacterial growth of ≥10^8^CFU/L and urine white cell count ≥10^8^/L and growth of an organism that is not candida.*

b *Antibiotics were based on the National Institute for Health and Care Excellence UTI antimicrobial guidelines and included trimethoprim, nitrofurantoin, amoxicillin, pivmecillinam, cefalexin, fosfomycin, ciprofloxacin, and co-amoxiclav.^20^^–^22CFU/L = colony forming units per litre. ICD = International Classification of Diseases. PEDW = Patient Episode Database for Wales. UTI = urinary tract infection. WLGP = Welsh Longitudinal General Practice. WRRS = Welsh Results Reporting Service.*

#### Definition of prophylactic antibiotics

Prophylactic antibiotic use was defined as ≥3 consecutive prescriptions for the same UTI-specific antibiotic (trimethoprim, nitrofurantoin, or cefalexin) with 21–56 days between prescriptions (Supplementary Box S5 and Supplementary Figure S2). This approach was required as WLGP data include prescribing data only (not dispensing) without data on the quantity of tablets prescribed. Women who used prophylactic antibiotics in the preceding 12 months were excluded to identify new users and ascertain urine culture results before initiation.

#### Comorbidity identification

Relevant comorbidities that either increase the risk of UTIs or potentially influence antibiotic prescribing were identified using Read codes and/or ICD-10 codes in the WLGP and PEDW data sources, respectively. To define these, the authors looked back from the date of cohort entry. The length of lookback was specific to the condition and further details are included in Supplementary Box S6.

#### Urine microbiology

Reported urine culture results were identified using code lists for urine tests in the WRRS (Supplementary Box S7). Analyses of urine specimens were restricted to 2015–2020 as there was a marked increase in NHS Wales laboratories submitting urine data from 2015.^23^ Urine culture results reported on the same day with the same result were regarded as duplicates. Urine microbiology results were categorised using a methodology based on the Public Health Wales Microbiology Division’s standard operating procedure using organism(s) cultured and white blood cell count (Supplementary Box S8).^24^ Antibiotic susceptibility was categorised using the European Committee on Antimicrobial Susceptibility Testing guidelines 2019, where intermediate is described as susceptible at increased exposure.^25^

In the study, all reported urine culture results in the 12 months before diagnosis (clinical cohort) and 18 months before prophylactic antibiotic initiation (prophylactic cohort) and all urine culture results within 7 days of an acute UTI were analysed to define the number of women with microbiologically confirmed rUTIs. The rationale for using 18 months before study entry for the prophylactic cohort was to account for potential delays between rUTI diagnosis and investigation or referral before initiating antibiotics.

### Statistical analysis

Sociodemographic and clinical characteristics were summarised using counts and percentages for categorical variables and means (with standard deviation) or medians (with interquartile range [IQR]) for continuous variables. Rates were calculated according to 10-year age bands using all women in SAIL aged ≥18 years and their person-time over the study period (2010–2020) as the denominator.

For women in both cohorts, the number of urine cultures before cohort entry, organisms cultured, and antibiotic susceptibility with subgroup analyses for *Escherichia coli* and coliforms are reported. The study also explored whether the proportions of urine cultures tested for susceptibility and the proportion that were resistant changed over the study period in view of changing incident prophylactic antibiotic use in the prophylaxis cohort.

For women in the prophylaxis cohort, antibiotic type and dose are also reported and how many met the definition of rUTIs was calculated. For those who did, time between meeting the rUTI definition and starting prophylactic antibiotics was calculated. Sensitivity analyses were conducted first restricting prophylactic antibiotic use to women on a consistent dose of antibiotics over consecutive prescriptions and secondly the definition used in this study for acute UTIs was adjusted to describe the proportion of women with rUTIs before starting prophylaxis to assess their impact on the estimates. Finally, how many women had a urine culture reported before initiating prophylactic antibiotics was estimated and the most recent result before initiation was used to ascertain their prophylactic antibiotic susceptibility.

Analyses were conducted in R version 4.1.3.26 The SAIL Databank Information Governance Review Panel approved the study. For analyses where counts are small, counts are rounded to the nearest 10 for the purposes of disclosure control and privacy protection. The Strengthening the Reporting of Observational Studies in Epidemiology (STROBE) checklist to guide reporting was used.^27^

## Results

**Figure 1.**
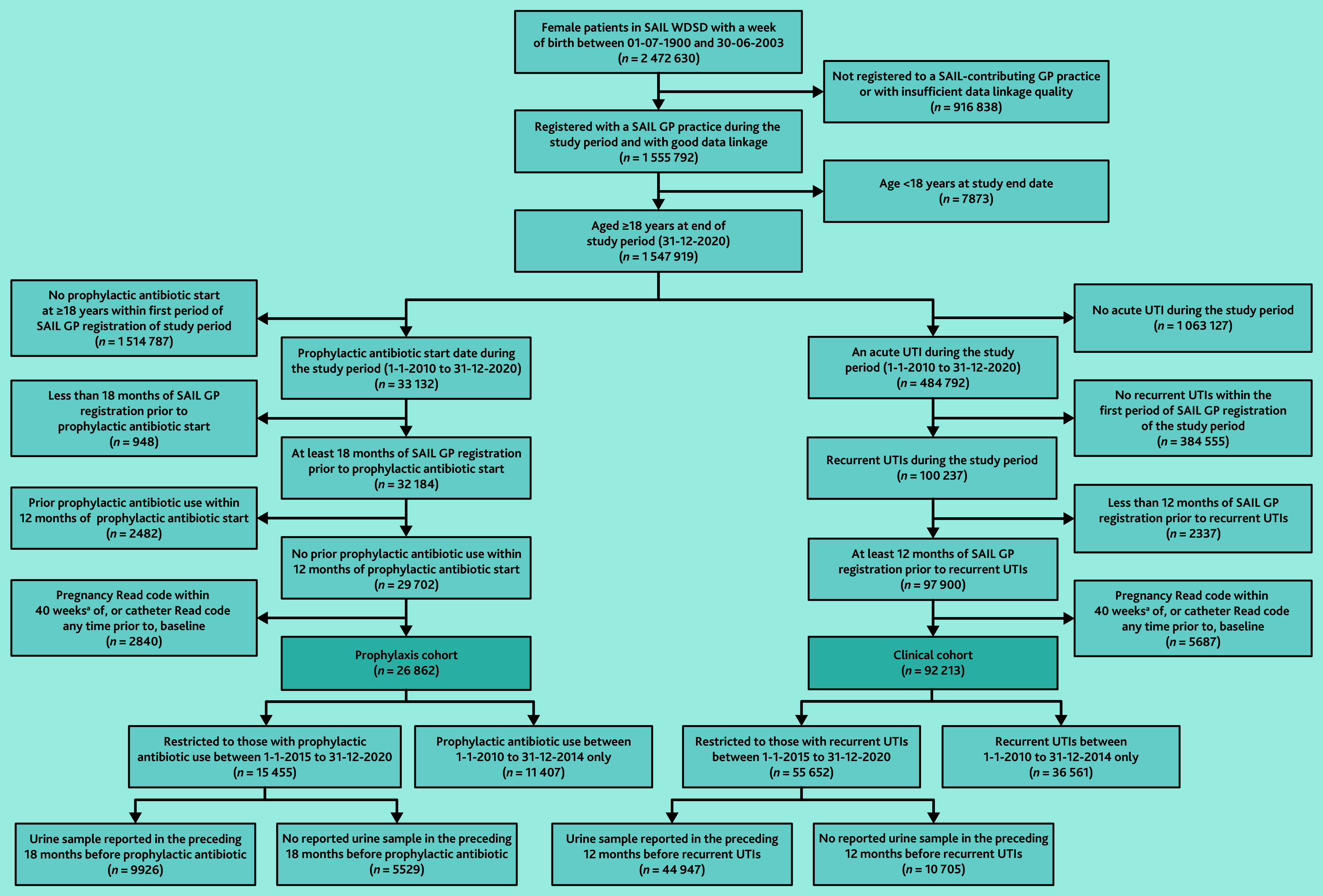
Flow charts for final study cohorts. aPregnancy Read codes recorded against individuals aged ≥55 years at time of pregnancy event are assumed to be a coding error and are therefore retained within the project cohort. SAIL = Secure Anonymised Information Linkage. UTI = urinary tract infection. WDSD = Welsh Demographic Service Dataset.

In total, 92 213 women (6.0% of women aged ≥18 years between 2010 and 2020) who met the clinical definition of rUTIs were identified and were entered into the clinical cohort (Figure 1), *N* = 1 547 919. Median age was 60.0 years (IQR 38.0–76.0).

**Figure 2.**
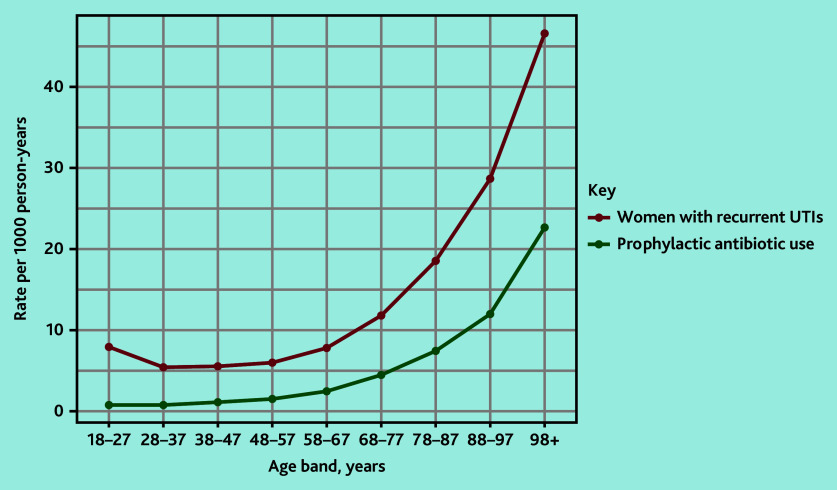
The rate of women with rUTIs and prophylactic antibiotic use over the study period according to age band. rUTI = recurrent urinary tract infection.

The rate of women with rUTIs followed a ‘J’ shaped pattern rising with increasing age (Figure 2).

The study identified 26 862 women (1.7%) with prescriptions for prophylactic antibiotics who formed the prophylaxis cohort (Figure 1), *N* = 1 547 919. Median age was 70.6 years (IQR 55.1–81.6). In all, 17 803 were included in both cohorts.

Prophylactic antibiotic initiation increased initially until 2012 before declining from 2013 (Supplementary Figure S3). Trimethoprim and cefalexin use declined with time, and nitrofurantoin use increased. The most used prophylactic antibiotic was trimethoprim (Supplementary Figure S4). A small proportion (<1.0%) of women appeared to be using multiple antibiotics concurrently. Most women were taking a consistent dose of the prophylactic antibiotic across the consecutive prescriptions (77.8%, *n* = 20 892/*N* = 26 862). The most prescribed dosages for trimethoprim were 100 mg and 200 mg, for nitrofurantoin were 50 mg or 100 mg, and for cefalexin were 250 mg and 500 mg.

The two cohorts were similar in terms of ethnic group and deprivation (Table 1). Most women with rUTIs were fit or had mild frailty according to the electronic frailty index, whereas for prophylactic antibiotic users all levels of frailty were higher (Table 1).^28^^,^^29^

**Table 1.** Sociodemographic and clinical characteristics of women with recurrent UTIs and prophylactic antibiotic users

**Cohort characteristic and level**	**Women with recurrent UTIs (clinical cohort)**	**Prophylactic antibiotic users (prophylaxis cohort)**
**Total cohort population, *n***	92 213	26 862
**Age, years, median (IQR)**	60.0 (38.0–76.0)	70.6 (55.1–81.6)

**Ethnic group, *n* (%)**		
White	51 446 (55.8)	15 526 (57.8)
All other ethnic groups combined	1368 (1.5)	241 (0.9)
Missing	39 399 (42.7)	11 095 (41.3)

**Deprivation quintile, WIMD, *n* (%)**		
1 (most deprived)	18 069 (19.6)	5064 (18.9)
2	17 790 (19.3)	5386 (20.1)
3	17 751 (19.2)	5133 (19.1)
4	16 896 (18.3)	4893 (18.2)
5 (least deprived)	18 339 (19.9)	5352 (19.9)
Missing	3368 (3.7)	1034 (3.8)

**BMI, median (IQR)^a^**	26.0 (23.0–31.0)	27.0 (23.0–31.0)

**Smoking status, *n* (%)**		
Never smoked	37 786 (41.0)	10 905 (40.6)
Ex-smoker	34 042 (36.9)	10 992 (40.9)
Current smoker	14 882 (16.1)	3423 (12.7)
Missing	5503 (6.0)	1542 (5.7)

**Alcohol status, *n* (%)**		
Non-drinker	32 996 (35.8)	11 485 (42.8)
Current drinker	43 611 (47.3)	12 118 (45.1)
Excess drinker	2419 (2.6)	604 (2.2)
Missing	13 187 (14.3)	2655 (9.9)

**Diagnosis location of UTIs contributing to recurrent UTI diagnosis, *n* (%)**		
General practice	76 233 (82.7)	N/A
Hospital	5490 (6.0)	N/A
Both	10 490 (11.4)	N/A

**Frailty score via electronic frailty index, *n* (%)**		
Fit	40 300 (43.7)	7070 (26.3)
Mild frailty	30 793 (33.4)	10 372 (38.6)
Moderate frailty	14 742 (16.0)	6495 (24.2)
Severe frailty	6378 (6.9)	2925 (10.9)

**Diabetes, *n* (%)**	20 410 (22.1)	7676 (28.6)

**Chronic kidney disease stage 3–5, *n* (%)**	12 827 (13.9)	5348 (19.9)

**Immunosuppression, *n* (%)**	4991 (5.4)	2060 (7.7)

**Urinary tract stones, *n* (%)**	412 (0.4)	258 (1.0)

**Urinary tract structural abnormality, *n* (%)**	1249 (1.4)	565 (2.1)

a *BMI was missing for 21 415 patients in the clinical cohort and 5411 patients in the prophylaxis cohort. BMI = body mass index. IQR = interquartile range. N/A = not applicable. UTI = urinary tract infection. WIMD = Welsh Index of Multiple Deprivation.*

### Clinical cohort

When the clinical cohort was restricted to those with rUTIs between 2015 and 2020, the cohort reduced to 55 652 women and 125 971 urine culture results. Of these 55 652, 44 947 (80.8%) women had a urine culture reported in the preceding 12 months, and, of these, 41.1% (*n* = 18 475/*N* = 44 947) had ≥3 samples reported. Of all urine cultures reported, 28.1% (*N* = 125 971) showed microbiological evidence of a UTI (Supplementary Figure S5) with *E. coli* the most cultured uropathogen (76.8% *n* = 41 987/*N* = 54 667) (Supplementary Figure S6). Based on urine culture results within 7 days of an acute UTI, 5.1% (*n* = 2866) had microbiologically confirmed rUTIs (that is, all UTIs contributing to the rUTI diagnosis were microbiologically confirmed).

Antibiotic susceptibility testing was low (under 40%) for most antibiotics except trimethoprim, nitrofurantoin, and amoxicillin. Trimethoprim and amoxicillin had high rates of resistance (Table 2).

**Table 2.** Antibiotic susceptibility for all urine culture results that cultured any organism, *Escherichia coli*, and coliforms

**Cohort**	**Susceptibility for urine culture results culturing any organism (clinical cohort *N* = 54 667, prophylaxis cohort *N* = 25 984)**		**Susceptibility for urine culture results culturing *E. Coli* (clinical cohort *N* = 41 987, prophylaxis cohort *N* = 19 669)**		**Susceptibility for urine culture results culturing a coliform (clinical cohort *N* = 50 119, prophylaxis cohort *N* = 23 784)**	

	**Proportion tested % (*n*)^a^**	**Resistance % (*n*)^b^**	**Proportion tested % (*n*)^c^**	**Resistance % (*n*)^b^**	**Proportion tested % (*n*)^d^**	**Resistance % (*n*)^b^**
**Clinical cohort**						
Trimethoprim	94.9 (51 878)	40.3 (20 918)	98.3 (41 255)	41.4 (17 065)	98.1 (49 151)	40.6 (19 949)
Nitrofurantoin	93.8 (51 277)	8.2 (4216)	97.4 (40 880)	2.9 (1200)	95.7 (47 962)	8.6 (4110)
Amoxicillin	82.1 (44 863)	57.1 (25 635)	82.8 (34 786)	56.8 (19 766)	83.4 (41 802)	60.5 (25 284)
Cefalexin^e^	23.6 (12 885)	15.8 (2042)	23.6 (9906)	14.6 (1443)	24.9 (12 466)	15.0 (1864)

**Prophylaxis cohort**						
Trimethoprim	95.3 (24 768)	44.6 (11 035)	98.7 (19 423)	46.5 (9036)	98.6 (23 456)	44.9 (10 526)
Nitrofurantoin	95.0 (24 695)	8.8 (2162)	98.2 (19 318)	3.2 (616)	96.9 (23 044)	9.2 (2126)
Amoxicillin	85.2 (22 126)	59.6 (13 195)	85.9 (16 900)	60.0 (10 137)	86.6 (20 593)	63.4 (13 060)
Cefalexin^e^	27.1 (7050)	14.5 (1022)	27.4 (5389)	13.4 (722)	28.7 (6837)	13.6 (929)

a *The denominator for the proportion tested is the total number of culture results that cultured any organism for that cohort, that is, 54 667 and 25 984 for the clinical and prophylaxis cohort respectively.*

b *The proportion of culture results with resistance to the respective antibiotics was calculated from the number of resistant cultures divided by the total proportion tested for susceptibility for that antibiotic.*

c *The denominator for the proportion tested is the total number of culture results culturing* E.coli *for that cohort, that is, 41 987 and 19 669 for the clinical and prophylaxis cohort respectively.*

d *The denominator for the proportion tested is the total number of culture results culturing a coliform for that cohort, that is, 50 119 and 23 784 for the clinical and prophylaxis cohort respectively.*

e *The resistance levels for cefalexin should be interpreted with caution since the proportions of urine cultures tested for susceptibility to cefalexin was low, suggesting selective testing.*

### Prophylaxis cohort

In the prophylaxis cohort, 49.0% (*n* = 13 149/*N* = 26 862) of women met the definition of rUTIs in the preceding 18 months. Of these, 39.1% (*n* = 5139/*N* = 13 149) started prophylactic antibiotics within 3 months of meeting the rUTI definition (Supplementary Figure S7).

When restricted to incident prophylactic antibiotic users between 2015 and 2020, the cohort reduced to 15 455 women and 53 988 urine culture results. Of these, 64.2% (*n* = 9926/*N* = 15 455) had ≥1 urine culture reported in the preceding 18 months and, of these, 77.0% (*n* = 7641/*N* = 9926) had ≥3 samples reported. Of reported urine cultures, 32.2% (*n* = 17 367/*N* = 53 988) were microbiologically confirmed UTIs, *E.coli* was the predominant organism (75.7%, *n* = 19 669/*N* = 25 984) cultured and 42.8% (*n* = 6611/*N* = 15 455) had at least one microbiologically confirmed UTI before starting prophylactic antibiotics (Supplementary Figures S8 and S9). Of women taking prophylactic antibiotics between 2015 and 2020, 49.8% (*n* = 7695/*N* = 15 455) had clinical rUTIs before initiation and, of these, 6.1% (*n* = 472/*N* = 7695) had microbiologically confirmed rUTIs. Like the clinical cohort, antibiotic susceptibility for trimethoprim and amoxicillin showed high rates of resistance (Table 2). However, the proportions of urine cultures growing any organism with evidence of resistance to trimethoprim or amoxicillin decreased over the study period (Supplementary Figure S10).

Based on the most recent urine culture that cultured an organism before initiating antibiotic prophylaxis, there were 4983 culture results. In women taking either trimethoprim, nitrofurantoin, or cefalexin (*n* = 4920), 8.3% (*n* = 410/*N* = 4920) had evidence of resistance to that antibiotic (Supplementary Table S1). This was highest in those taking trimethoprim (18.5%, *n* = 320/*N* = 1730), with a downward trend over the study period (Supplementary Figure S11). Resistance was lower for those taking nitrofurantoin (which was consistent over the study period) and cefalexin (trend not shown because of small numbers) (Supplementary Figure S11 and Supplementary Table S1). Resistance to these three prophylactic antibiotics, irrespective of which antibiotic was prescribed (including those taking multiple antibiotics), was 41.5% (*n* = 2070/*N* = 4983) for trimethoprim, 6.4% (*n* = 320/*N* = 4983) for nitrofurantoin, and 3.0% (*n* = 150/*N* = 4983) for cefalexin.

Sensitivity analyses only defining prophylactic antibiotic use if the dose prescribed was consistent across consecutive prescriptions did not meaningfully affect the estimates (Supplementary Table S2). Changing the minimum time between acute UTIs and changing the definition of an acute UTI to include Read codes only did not meaningfully have an impact on the estimate of women with rUTIs before starting prophylaxis (Supplementary Table S3). Changing the acute UTI definition used to include only UTI-related antibiotics did increase the proportion of women with rUTIs before starting prophylaxis to 74.3% (*n* = 19 970/*N* = 26 862).

## Discussion

### Summary

This is the first population-based study, to the authors’ knowledge, to describe the prevalence of rUTIs, prophylactic antibiotic use, and associated microbiology in women in the UK. The current study found that, of women registered with a SAIL data-providing general practice in Wales between 2010 and 2020, 6.0% had rUTIs, and 1.7% were prescribed prophylactic antibiotics with the proportions rising sharply around 58–67 years of age. Nearly half of users of prophylactic antibiotics met the rUTI definition in the 18 months before initiation, and initiation of prophylactic antibiotics decreased over the study period.

In total 80.8% of women with rUTIs had a urine culture reported in the 12 months before the diagnosis with high levels of resistance to trimethoprim and amoxicillin. Microbiological evidence of a UTI was present in 28.1% (*n* = 35 404/*N* = 125 971) of all reported urine cultures. Urine culture before initiating prophylactic antibiotics was reported in 64.2%. Of women prescribed trimethoprim, 18.5% had evidence of resistance to it before initiation. As part of rUTI diagnosis and before initiating prophylactic antibiotics, more frequent urine cultures could better inform antibiotic choice for prophylaxis and treatment.

### Strengths and limitations

This study used a large population-based sample to identify women with rUTIs and prescribed prophylactic antibiotics with linked urine microbiology including all urine culture within NHS Wales. The authors comprehensively reported urine microbiology results and resistance patterns. This study population was representative of women in the wider Welsh population and women with and without adequate lookback data had similar characteristics^14^ (Supplementary Figures S12–S17). A conservative estimate of rUTI prevalence is likely both from using a 28-day window to avoid capturing UTI-relapse and not having data from out-of-hours general practice or hospital attendances not requiring admission. UTI-related codes were used to identify acute UTIs but if clinicians had used non-specific codes these UTIs would not be captured, again underestimating rUTI prevalence.

As a result of these potential limitations, when describing the proportion of women with rUTIs before prophylactic antibiotic initiation, the authors adjusted both the minimum time between UTIs and the definition of an acute UTI to include only UTI-specific Read codes (and potentially capture UTIs diagnosed in out-of-hours general practice or hospital attendances) or to include only antibiotics in sensitivity analyses. Changing the time between UTIs and defining an acute UTI based on UTI-specific Read codes had a minimal impact (Supplementary Table S3). Changing the definition of an acute UTI to include only antibiotics, increased the proportion with rUTIs but likely overestimated the true value (Supplementary Table S3).

The data used in the current study are primarily used for clinical practice, not research, with risks of coding errors, missing data, and misclassification. Misclassification of prophylactic antibiotics is possible where the women the authors defined as users of prophylactic antibiotics could have had three acute antibiotic courses. A variety of methods were used to reduce this risk, such as using a fixed timeframe between prescriptions and conducting a sensitivity analysis to assess the robustness of the estimates. Finally, the diagnosis of UTIs is especially challenging in older, frailer women where symptoms can be less specific, and they may have asymptomatic bacteriuria and thus be misdiagnosed as having a UTI. This could potentially falsely elevate the prevalence of rUTIs; however, the authors used UTI-specific Read codes in addition to antibiotic prescriptions to try to mitigate for this.

### Comparison with existing literature

To the authors’ knowledge there is only one other study using population-based data describing women with rUTIs.^30^ This US-based study identified women with incident rUTIs and found 60.9% of women had at least one urine culture over 12 months before diagnosis, lower than the proportion found in the current study. The current study cohort likely includes both incident and prevalent rUTIs as the authors did not stipulate a UTI-free period before rUTI cohort entry. Therefore urine culture, as per guidelines, might be more likely to occur in the current cohort in those with prevalent rUTIs. The US-based study also found a ‘J’ shaped curve for the incidence rate of rUTIs according to age. This likely relates to UTI risk factors such as sexual intercourse in early adulthood^5^^,^^31^ and hormonal changes of the menopause accounting for the increased rate at about 55 years of age (the typical age of menopause is between 45 and 55 years).^31^^–^35 In terms of rUTI prevalence, the current results suggest the prevalence is higher than that of a survey conducted in 2015 that found a prevalence of 3%.^8^ This is not surprising as certain populations such as frail women or those with cognitive impairment may not complete a survey, whereas they are more likely included in the current study’s cohort.

Prophylactic antibiotic use in the current study declined from 2013 to 2020. Overall antibiotic prescribing followed a similar pattern in both Wales and England, aligning with the UK Government’s strategy to reduce antibiotic use and combat increasing AMR.^36^^–^38 Resistance in UTIs is an increasing problem and evidence of UTI resistance patterns in women with rUTIs is limited. The current study shows that trimethoprim and nitrofurantoin resistance in women with rUTIs are comparable with those reported in a 2018 and 2023 Public Health Wales report.^39^^,^^40^ This suggests resistance in women with rUTIs is not significantly higher than resistance patterns overall.

### Implications for practice

It could be clinically beneficial to encourage microbiological confirmation of rUTIs in primary care and before prophylactic antibiotic initiation in line with clinical guidelines.^5^^,^^31^ Women with rUTIs in the current study had high levels of resistance to trimethoprim and amoxicillin, which are two of the four prophylactic antibiotics recommended in the UK.^31^ A low proportion of urine cultures reported susceptibility to cefalexin, and although resistance levels were relatively low they should be interpreted with caution because of likely selective testing. Despite low resistance levels, nitrofurantoin has limitations in chronic kidney disease and can result in lung and liver fibrosis with the risk increasing with age and duration of use.^41^^–^43 The current study has also shown that 18.5% of women prescribed trimethoprim had evidence of resistance before initiation. These findings emphasise urine culture’s potential importance in informing prophylactic antibiotic choice. However, increasing urine culture has limitations owing to negative culture results, culturing a mixed growth of organisms, or the initiator, in primary or secondary care, not having access to all recent urine microbiology results.

In conclusion, this is the first population-based study on rUTIs and prophylactic antibiotic use in women including urine microbiology. The prevalence of rUTIs in women and the incident use of prophylactic antibiotics in Wales, although declining with time, was substantial especially in older women. Women with rUTIs had high levels of resistance to two of the four recommended prophylactic antibiotics, 64.2% had urine culture before starting prophylactic antibiotics, and a significant proportion had evidence of resistance to that antibiotic.

## Data

The data used in this study are available in the SAIL Databank at Swansea University, Swansea, UK, but, as restrictions apply, they are not publicly available. The code for preparing the data in SAIL and the analytical R code are available on reasonable request to the lead author.
